# Risky behaviours and their correlates among adolescents living with HIV in sub-Saharan Africa: a systematic review

**DOI:** 10.1186/s12978-018-0614-4

**Published:** 2018-10-24

**Authors:** Maggie Zgambo, Fatch Welcome Kalembo, Balwani Chingatichifwe Mbakaya

**Affiliations:** 1 St John’s College of Nursing and Midwifery, P.O Box 18, Mzuzu, Malawi; 2 Faculty of Health Sciences, Mzuzu University, Private Bag 201, Luwinga, Mzuzu 2, Malawi

**Keywords:** Adolescents, Health behaviour, Risky behaviour, Living with HIV and AIDS, HIV disclosure, ART adherence, Correlates, Sub-Saharan Africa

## Abstract

**Background:**

Adolescents living with HIV (ALWHIV) in sub-Saharan Africa encounter multiple health problems that are often unrecognised by the public and the healthcare workforce. The aim of this systematic review was to identify risky health behaviours and their associated factors among ALWHIV in sub-Saharan Africa.

**Methods:**

We systematically searched for articles in Medline, SCOPUS, Directory of Open Access Journals, Science Direct, ProQuest, Psych-info, Web of science, WHO Global Index Medicus library, Cochrane, and Google Scholar. Studies were included in this review if: they were original studies; participants were aged from 10 to 19 years; participants were ALWHIV or they had data from different key informants focusing on ALWHIV within the age group; they had health behaviours as an outcome; they were conducted in sub-Saharan Africa and were published before December 2016. Data were extracted and the quality of the studies was appraised using the Mixed Method Appraisal Tool (MMAT).

**Results:**

Thirty-six studies met the eligibility criteria. Nineteen studies scored 100% (indicating high quality), sixteen studies scored 75% (indicating moderate quality) and one study scored 50% (indicating low quality) on the MMAT scale. Adherence to antiretroviral therapy among ALWHIV was suboptimal and was negatively affected by forgetfulness, opportunistic infection, long distance to clinics, and fear of unplanned disclosure. Many adolescents were sexually active, but the majority did not disclose their HIV status to sexual partners, despite knowing their diagnosis (range 76–100% across available studies) and some did not use protection (condoms) to prevent transmission of HIV and other sexually transmitted diseases (range 35–55%). Disclosure to and from adolescents was low across the studies and was associated with fear of disclosure aftermaths including stigma and discrimination (range 40–57%).

**Conclusion:**

A considerable proportion of ALWHIV in sub-Saharan Africa engage in multiple risky health behaviours, which have a substantial negative impact on their wellbeing and cause significant risk and burden to their families, sexual partners and societies.

**Electronic supplementary material:**

The online version of this article (10.1186/s12978-018-0614-4) contains supplementary material, which is available to authorized users.

## Plain English summary

HIV/AIDS continues to be a major problem in sub-Saharan Africa and adolescents in this region have not been spared from this pandemic disease. Although the needs of adolescents living with HIV (ALWHIV) are given less attention, they display behaviours that pose a risk to their health, others and are a potential burden to society and nations. This article summarises research on ALWHIV in sub-Saharan Africa, with an aim to determine the risky health behaviours displayed by ALWHIV aged from 10 to 19 years, prevalence rates and the associated factors. Only research articles in scientific databases that met our specified criteria were included in this review. We identified and included 36 scientific articles. We found that adolescents lag in adherence to HIV medicine with rates (reported in articles) as low as 6.6%. The reasons for this included forgetfulness, physical health, distance from clinics, religion, and fear of unplanned disclosure. Also, it was noted that parents find it difficult to disclose to their children that they (ALWHIV) are HIV positive. Some adolescents find it impossible to disclose their HIV status to their sexual partners and others, despite being sexually active. Among the sexually active adolescents, condom use was reported to be low (less than half) on their first and last time they had sex. We conclude that behaviours displayed by ALWHIV in sub-Saharan Africa pose a significant risk and burden to their families, sexual partners, and societies.

## Background

HIV continues to have devastating effects on adolescents in sub-Saharan Africa (SSA), despite decades of treatment and care. In 2015, there were approximately 1.8 million adolescents (10–19 years old) living with Human Immunodeficiency Virus (HIV) in SSA, which accounted for 80% of the global HIV infections in adolescents [1]. Although HIV-related deaths are reportedly decreasing in other age groups, they are rising among adolescents, and they are predominant in the African region [1, 2]. Acquired Immune Deficiency Syndrome (AIDS) is now the number one cause of death among adolescents in Africa, accounting for one in every six adolescent deaths [1, 3]. While the rate of HIV transmission is decreasing in the general population in SSA, new infections among adolescents are increasing [1, 2, 4].

Even though the majority of adolescents living with HIV (ALWHIV) have acquired the virus through mother to child transmission (MTCT), a small proportion contracted the virus through unprotected sex or injectable drugs [5]. Advances in treatment have increased the survival rate of ALWHIV who acquired HIV through MTCT such that they are now entering early adulthood [3, 6]. Although the World Health Organisation (WHO) recommends that children should be told about their HIV status by the age of 12 years [7], recent studies conducted in SSA indicated a low prevalence of HIV disclosure to ALWHIV [7–9]. Moreover, despite decades of increased coverage of HIV campaigns, 36% of boys and 30% of girls have no comprehensive knowledge of HIV prevention [7]. The most worrisome fact is that fewer than one-third of adolescents aged 15–19 years, who have multiple sexual partners, report having used condoms during sexual encounters [7]. While the lifespan of ALWHIV in SSA has increased with anti-retroviral therapy (ART), their life expectancy is still far below their counterparts in resource-rich settings [10–14]. ALWHIV in resource-rich countries such as Australia and the Netherlands are expected to have an average lifespan similar to a person without HIV [12]. Moreover, the viral suppression (undetectable HIV ribonucleic acid), which is an indicator of the effectiveness of antiretroviral medication, is low among adolescents in many SSA countries compared to adolescents in resource-rich countries [13, 15, 16]. In addition, the prevalence of mental health problems among adolescents in SSA is higher among ALWHIV compared to those without the virus [17–21].

Despite the growing research interest in ALWHIV, little evidence exists on understanding risky and other important health behaviours of this group in the SSA region. Scientific evidence about ALWHIV provides a framework for developing policies, guidelines and interventions that can prevent and curb the escalation of challenges and problems encountered by these adolescents, caregivers, and health workers. The number of HIV-surviving adolescents (those who have lived with HIV from birth) and their life expectancy are increasing, which requires an understanding of the experiences, challenges, needs, their sexuality, ART adherence and HIV social disclosure. A systematic review was needed to document the evidence on risky health behaviours and their correlates among ALWHIV in SSA. The review addresses two questions: 1) What risky health behaviours do ALWHIV in SSA engage in? and, 2) What are the factors associated with these behaviours among ALWHIV in SSA?

## Methodology

The protocol for this review was registered in PROSPERO (registration number CRD42016051392) [22]. The Mixed Method Appraisal Tool (MMAT) was used to appraise the quality of included studies.

### Inclusion criteria

Irrespective of study design, articles were included in this review if they: (a) were original studies; (b) restricted participants to those aged from 10 to 19 years (for studies that included youth within this age group and older, we extracted data for those younger than 20 years if they were reported separately); (c) had study participants who were ALWHIV; (d) focused on ALWHIV within the age group; (e) had predefined cohorts of ALWHIV within the age group; (f) had risky health behavioural outcomes (primary or secondary) in line with our objectives; (g) were conducted in SSA; and (h) were published in English from 1983 to December 2016.

We defined an adolescent as a person aged from 10 to 19 years old [23]. We defined risky health behaviours as behaviours that have negative long- or short-term consequences on the health of an individual. We used the Centre for Disease Control and Prevention (CDC) list of risky behaviours as a point of reference for identifying risky behaviours among ALWHIV [24]. The CDC outlines the following as priority risky health behaviours among youth: behaviours causing violence and bodily harm, unprotected sex leading to unintended pregnancy and sexually transmitted infections (STIs), alcohol and other drug use, tobacco use, unhealthy dietary behaviours, and inadequate physical activity [24]. In this review, we also included ART non-adherence as a risky health behaviour. Lack of HIV disclosure to an adolescent living with HIV or their significant people like sexual partners, teachers and relatives (social disclosure) was included as a secondary outcome. We did not include psychological and health system related behavioural outcomes and associated factors as these areas are vast and need distinct investigation elsewhere.

### Search strategy

We searched the following databases: The Medline, SCOPUS, Directory of Open Access Journals, Science Direct, ProQuest, Psych-info, Web of science, WHO global index medicus library, Cochrane, and Google Scholar databases. The following key search terms were used: Adolescent OR youth OR young adult OR children; AND Living with HIV OR HIV infections OR HIV seropositivity OR HIV OR HIV OR hiv1 OR HIV 2 OR human immunodeficiency virus OR AIDS OR human immunodeficiency disease; AND Antiretroviral therapy adherence OR adherence OR medication adherence OR patient compliance OR highly active antiretroviral therapy OR anti-retroviral agents; OR risk factor OR risky behaviour OR unsafe sex OR sexual behaviour OR risk-taking OR alcoholism OR alcohol drinking OR smoking OR attitude OR practice OR health knowledge OR adolescent health OR adolescent behaviour; OR HIV disclosure OR truth disclosure; AND Africa OR sub-Saharan Africa OR South of the Sahara OR South Africa OR Africa Central OR Africa Eastern OR Africa Western OR Africa Southern. In addition, we also conducted a separate search of articles using the search terms above for each of the SSA countries including Angola OR Benin OR Botswana OR Burkina Faso OR Burundi OR Cameroon OR Cape Verde OR Central African Republic OR Chad OR Comoros OR Congo Brazzaville OR Congo Democratic Republic OR Côte d’Ivoire OR Djibouti OR Equatorial Guinea OR Eritrea OR Ethiopia OR Gabon OR The Gambia OR Ghana OR Guinea OR Guinea-Bissau Or Kenya OR Lesotho OR Liberia OR Madagascar OR Malawi OR Mali OR Mauritania OR Mauritius OR Mozambique OR Namibia OR Niger OR Nigeria OR Rwanda OR Senegal OR Sierra Leone OR Somalia OR South Africa OR Sudan OR Swaziland OR Tanzania OR Togo OR Uganda OR Zambia OR Zimbabwe. An additional table illustrates the search stratergy further [see Additional file 1]. Furthermore, keywords such as, risk factors, barriers, facilitators of living with HIV, experiences, predictors and correlates were also combined using Boolean operations OR and AND. Efforts were also made to identify both published and unpublished interventional studies by manually searching conference proceedings such as, the International AIDS Conference, International AIDS Society (IAS) Conference on HIV Science, and The Annual Conference on Retroviruses and Opportunistic Infections.

### Study selection

After retrieving the articles from the databases, they were imported to Endnote X7 Reference Management System. The titles and abstracts of the retrieved articles were merged in Endnote and duplicates were removed. Finally, the title, abstract and full articles were reviewed against the inclusion criteria.

### Data extraction

**Fig. 1:**
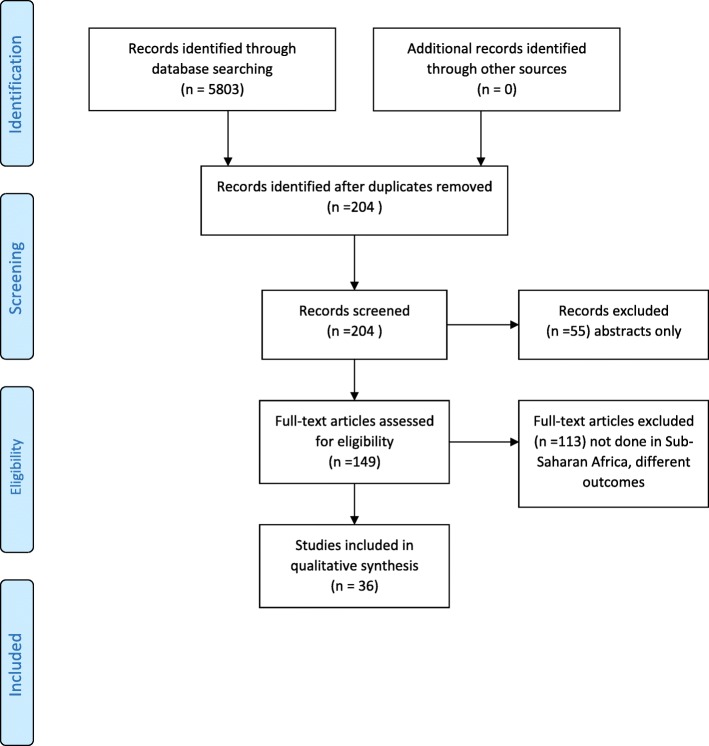
PRISMA Flow Diagram

Data were extracted manually following the preferred reporting items for systematic reviews and meta-analysis (PRISMA) guidelines as illustrated further in the shown in Fig. 1 (Prisma Flow Diagram) [25, 26]. After the database search, MZ and BCM separately identified articles that potentially met the inclusion criteria for this review and screened them independently for eligibility. Lack of consensus over the eligibility of a particular article by the two reviewers was resolved through discussion with FWK. A standardised table was used to extract data from the included articles to assess the quality of the studies and to synthesise evidence. The details of the data extracted from the studies included: author, year of study, type of participants, the age of participants, setting, country, sample size, study design, methods, study purpose, study objectives, primary or secondary outcomes, and associated factors. Extraction of relevant information was summarised, documented and illustrated in detail [see Additional file 2 and Additional file 3].

### Quality appraisal

Using the MMAT tool, three authors independently assessed the risk of bias in the included studies by considering the clarity of the research questions (objectives) and whether the collected data addressed the research questions. In addition, quantitative studies were assessed if there was a complete outcome data (80% or above) and, when applicable, an acceptable response rate (60% or above). The MMAT is a checklist that includes specific criteria for assessing qualitative studies (4 criteria), randomised controlled trials (4 criteria), non-randomized studies (4 criteria) and mixed methods studies (3 criteria). Each criterion is worth 25% and summed, with a maximum score of 100% per study (in the case of mixed method studies, the first 25% is given by default then added to the scores of the three criteria).

### Synthesis of results

This systematic review identified behaviours and their associated factors among ALWHIV in SSA. A subset analysis categorised data into sexual risk behaviours, ART non-adherence, and HIV disclosure. A narrative synthesis was completed with a content analysis of the findings. The included articles were synthesised, rated for quality and the results were entered into a table [see Additional file 2 and Additional file 3].

## Results

### Search outcome

The search yielded 5,807 articles and 5,603 papers were excluded in a preliminary assessment stage of abstract and title. Two hundred and four articles were thoroughly assessed in full and only 36 met the inclusion criteria. Most of the identified articles were rejected from this systematic review because they did not meet our inclusion criteria on age, outcomes or region. The flow of how articles were included and excluded in this review is illustrated in detail in the given figure (see Fig. 1).

All the 36 studies were conducted between 2009 and 2016. Twenty-eight studies were carried out between 2011 and 2016. Eight studies were conducted in South Africa [27–34], seven in Uganda [35–42], four in Zambia [43–46] and four in Kenya [47–50]. Two studies were completed in each of the following countries: Zimbabwe [51, 52], Malawi [18, 53], Botswana [54, 55] and Ghana [56, 57]. One study was conducted in both Uganda and Zimbabwe [58] and another study was conducted in both Uganda and Kenya [41]. Two studies were conducted each in Côte d’Ivoire [59] and Nigeria [60]. Another study by Nachega et al. (2009) indicated that it was undertaken in Southern African countries, but the exact countries in the southern African region were not specified [61]. Among the 36 included studies 17 were qualitative [18, 27, 28, 33, 34, 40, 43–47, 52–54, 56–58], 12 were quantitative [29, 31, 35, 37, 38, 41, 48, 50, 55, 59–61], and seven were mixed methods [30, 32, 36, 39, 49, 51, 57]. Overall, 28 were cross-sectional studies [18, 27–30, 32–36, 39–41, 43–54, 56, 57], six were cohort studies [37, 55, 58–61] and two were randomised controlled trials [31, 38]. With regard to the quality of studies reported in the articles, 19 studies scored 100%, 16 studies scored 75% and one study scored 50% on MMAT scale.

Data were collected through interviews (structured, semi-structured or in-depth interviews) and focus group discussions as illustrated further in a table [see Additional file 3]. The quantitative studies had a response rate ranging from 97 to 100%. Studies were analysed according to four behavioural outcomes and their correlates, namely: risky sexual behaviours, ART non-adherence (any level of ART non-adherence), alcohol and substance abuse and HIV disclosure (HIV disclosure to or from adolescents). Authors of the articles described these outcomes in two parameters and information was extracted, analysed and grouped accordingly under each outcome. These parameters were the behaviours identified and their prevalence, and factors associated with the behaviours (correlates or protective factors according to retrieved information).

### Study participants

The mean age range of adolescent participants enrolled in these studies ranged from 12 to 17 years and the majority of the studies had participants with an age range of 10 to19 years old. This is demonstrated further in a table [see Additional file 3]. The number of participants in each study varied from 18 [54] to 1,059 [29].

### Behavioural outcomes

#### Risky sexual behaviours

The authors of the studies reported that the proportion of ALWHIV engaging in sexual activities of some form including sexual intercourse, ranged from 14.9 to 64% [28, 30, 35, 36]. One study found that 89% and 63% of males and females respectively had had sex already while another study found that 57% of the 34% who already had had sexual intercourse were females and 10% of these had it before the age of 15 years [35, 36]. Furthermore, 53% and 44% of participants who engaged in sexual activities in two different studies had never used any form of contraception [34, 36]. Moreover, condom use was found to be inconsistent (up to 76%) among those who reported to be using contraception [35, 36]. In addition, 24%, 33% and 85% of the participants in one South African [30] and two Ugandan [35, 42] studies respectively, had sexual partners, either a boyfriend or girlfriend. Nonetheless, another group of ALWHIV (41%) did not engage in sexual activities because of their HIV status, but others (8%) chose to have a partner who was also HIV positive [36]. Arikawa and colleagues further found 17 incidents of pregnancies in their 266 female ALWHIV sample in Côte d’Ivoire with two recurring [59]. Similarly, Birungi and colleagues (2011) found that 74% of the 506 pregnancies among 393 ALWHIV in their longitudinal study occurred unintentionally and that husbands were responsible for only 25% of these pregnancies [59]. Authors of two studies conducted in Côte d’Ivoire and Kenya reported that 28.5% and 10% of pregnancies respectively among ALWHIV resulted in abortion [48]. Median age at first pregnancy was 17.7 years in one study; another found that 79% of pregnancies occurred among adolescents who had first pregnancies before the age of 18 years. Likewise, Obare and colleagues (2012), found that 76% of the pregnancies in their study occurred to adolescents who experienced their first pregnancies before the age of 17 years [59] and mostly increased with age [39, 48, 49, 59].

##### Protective factors of risky sexual behaviours

ALWHIV in two studies mentioned the following as reasons for not having sex; fear of infecting others, not ready for sex, discouraged by health workers and guardians, fear of unwanted pregnancy, waiting until an older age, fear of sex and feeling that premarital sex is wrong [33, 36]. It was further reported that more male than female ALWHIV had had sexual intercourse (*p* < 0.005) [36, 49]. In contrast, authors of two other studies reported that female ALWHIV were more likely to have had sexual intercourse than their male counterparts (OR = 1.71, 95% [CI =1.21–2.42] *p = .001*) [30, 36]. Notably, older adolescents (18–19 years) than younger ones (15–17 years) were associated with ever engaged in sex (*p* < .05) [49]. Similarly, another study found that older ALWHIV (15–19 years) were associated with ever had sexual intercourse compared to younger participants aged from 10 to 14 years (OR = 12.38, 95% [C *I* = 5.34–28.7] *p = .001*) [35]. Although females in one study were reported to have a higher mean age (about 16 years old) at sexual debut than males (about 14 years old) (*p* < .001), males were reported to have higher percentile of using condoms or abstaining from sex than females (*p* < .05) [30, 49]. Additionally, use of contraception was positively associated with being married or living together (*p* < .005) [35] and knowing HIV status (*p* < .05) [36, 39, 60]. In one study, it was found that ALWHIV who knew their HIV status were likely to report that they had ever used a protective method at first intercourse (*p* ≤ .05), a contraceptive method (*p* ≤ 0.05), a modern method of contraception (*p* ≤ .01), and condoms (*p* ≤ .01) than their counterpart who did not know their status [39]. Also, knowing one’s HIV status was associated with safe sex (OR = 4.36, CI [1.09–17.47], *p* = .038), but disclosure did not improve sex negotiation as ALWHIV were still anxious with HIV disclosure [30]. Conflicting findings from a study in Nigeria were that ALWHIV (48.2%) who knew their HIV status engaged in unprotected sex (*p* = .0001) compared to 6% of those who did not know their HIV status and more ALWHIV who knew their status were having multiple `sexual partners (*p* = .0001) [60]. On the other hand, ALWHIV who reported to have had sex without protection (condoms) did so because of trust culminating from the longtime relationship and abuse (in a sexual relationship [33, 36]. In addition, the ALWHIV with one sexual partner were more likely to present with better mental health (OR: 1.94, 95% CI [1.00–3.74]) compared to those with two or more sexual partners [35]. Similarly, ALWHIV who had a boyfriend or girlfriend in one study were found to have better mental health status than their counterparts who were not in a sexual relationship (*p* = .02) [18].

Furthermore, sexual partners other than husbands were more likely to be responsible for unintended pregnancies (*p* < .01) as found in one study [48]. The authors of this study also found that pregnancies were significantly less likely to result in adverse outcomes if they occurred within marital unions (OR: 0.1, 95% CI [0.1–0.2], *p* < .05) [48]. However, some ALWHIV believed that couples who are HIV positive need not conceive but rather adopt children should they need them [33]. In addition, pregnant female ALWHIV were significantly shunning the prevention of mother to child transmission (PMTCT) services than regular prenatal services (67% vs. 84%; *p* < .01), [49]. In one study, girls desired to learn more about pregnancy but reported lack of sufficient reproductive health services and they feared or felt embarrassed to seek advice on sexual matters because it could be interpreted that they are sexually active or planning to [34]. Some participants believed that both parents and health workers gave them wrong information to deter them from sexual activity [34, 43]. Consequently, some adolescents opted for peers, media and internet for sexual and reproductive health information [34].

##### ART non-adherence

Self-reported ART adherence was suboptimal among adolescents, albeit known to be important in this population and the ART adherence rate ranged from 6.6 to 71% [29, 37, 49, 51, 54, 61]. Missing ART doses by participants were reported in four studies with a range from 18 to 62.5% [29, 44, 50, 61]. One study went further to compare ART adherence between ALWHIV and adults at different intervals and found that fewer adolescents achieved a 100% adherence at each time point stipulated than adults [61].

#### Correlates of ART adherence

Poor social-economic factors were widely reported as barriers to ART adherence [32, 37, 40, 56]. Cluver et al. (2015) found that ART non-adherence was associated with longer distance to the clinics (OR 0.54, 95% CI [0.39–0.73], *p* < .001) [29]. These findings are similar to those reported by authors of another study who found that the respondents living close to the clinic had greater odds of optimal adherence (OR 1.49, 95% CI [1.02–2.18], *p* < .05) [37]. In addition, lack of financial resources was identified as a reason that deterred some YLHIV from visiting HIV clinics to collect their medication [40, 56]. On the other hand, authors of a cross-sectional study conducted in South Africa found that sufficient food was significantly associated with optimal ART adherence (aOR 0.57, CI [0.42–0.76], *p* < .001) [32]. Surprisingly, even resources like material possession and employment were reported to affect ART non-adherence. In one study, higher asset possession and higher score of assets and employment composite measure were significantly associated with optimal ART adherence (OR 1.69, 95% CI [1.00–2.85], *p* < .05) and (aOR 1.70, 95% CI [1.07–2.70], *p* < .05) respectively [37].

Moreover, adolescents’ knowledge of their HIV status doubled the odds of past-week full adherence (OR 2.18; 95% CI [1.47–3.24], *p* < .001) [29, 52]. Although knowledge of HIV status increased with age, disclosure before the age of 12, was found to associate with higher ART adherence (OR 2.65; 95% CI [1.34–5.22]*, p < .*005) [29]. Likewise, the impartation of knowledge on HIV/AIDS assisted the participants to talk about their drug adherence slippages during support group meetings [52]. Support groups were found to have a positive impact on adherence in few studies [32, 40, 51]. Attending a support group was significantly associated with optimal ART adherence (aOR 0.60, 95% CI [0.40–0.91], *p* < .05) [29]. However, the notable support for adherence also included support from health worker and parents who directly encouraged the adolescents to take their ART or scolded them for missing doses, which negatively reinforced adolescents to comply with their ART regimes [40, 44, 56]. Moreover, high parental supervision/ monitoring was associated with ART adherence in one study (aOR 0.56, 95% CI [0.43–0.73], *p* < .001) [29]. In other studies, younger ALWHIV depended on caregivers for ART adherence [29, 32, 44, 47]. Additionally, another study found that adherence was relatively lower among ALWHIV than adults but did not specify the age groups of adolescents [61]. Intrinsic factors like the desire to live longer and take drugs every day [44], self-motivation [56], and their perceived positive outcomes [44, 56] were also mentioned to facilitate ART adherence. Other highlighted pharmacodynamical factors that hampered adherence were adverse effects of ART [40, 44], dispensed formula [43, 56] and opportunistic infections [29, 32]. Finally, forgetfulness [56], perceived stigmatisation [56], fear of unplanned disclosure [40, 44, 54], travelling [44] and lack of time [40] were reported to promote ART non-adherence [44]. However, adolescents who had started ART presented with better mental health (AOR = 3.9, 95% CI [2.22–6.92] *p* = .001) than those who were not on ART [35].

##### Alcohol, substance abuse and protective factors

Few studies explored the relationship between ALWHIV and substance abuse. Alcohol and tobacco were the two substances mentioned in three studies. It was found that non-smoking ALWHIV were nearly twice likely (OR 1.70, 95% CI [1.09–2.74] *p* = .001) to present with better physical and mental health than those who were smoking [35]. In one study, 2.7% of ALWHIV reported having taken alcohol at least once for the past 30 days before the study was conducted [18]. The researchers associated self-reports of alcohol consumption and good mental health (*p* = .040) [18]. ALWHIV in one randomised controlled trial were exposed to cognitive behavioural therapy (CBT) group counselling intervention weekly for eight consecutive weeks and alcohol consumption decreased in both interventional and control groups at post-test {interventional arm; 0.69 (*SD* = 2.73) to 0.26 (*SD* = 1.55), and the control group; 0.82 (*SD* = 3.82) to 0.47 (SD = 2.57)} [38]. In this study by Senyoni et al. (2012), alcohol consumption was self-reported and measured with the alcohol use disorders identification test [38].

##### HIV disclosure

HIV disclosure in the studies was looked at in two different ways, the first one being HIV status disclosure from ALWHIV to significant others, secondly, disclosure by the caregiver or health worker to the ALWHIV. Rates among ALWHIV who knew their HIV status in these studies ranged from 62.2 to 70% [30, 32, 60]. Surprisingly, good numbers of the participants in the studies who knew their HIV status had not yet disclosed to others with rates ranging from 13.5 to 75% [27, 28, 30, 36, 41]. Percentiles of those who disclosed their HIV status to friends were 10.5% and 17% in two studies [27, 41]. Authors in one study indicated that 34% of their participants had talked about the HIV diagnosis in sexual relationships. Likewise, only 35.5% of ALWHIV had disclosed their HIV status to their partners and 41.5% of those who were sexually active knew the status of their partners [30].

Secondly, ALWHIV mostly learned about their HIV status from their caregivers or the health workers. Authors of one study found that 72% of their participants were disclosed to by the health workers after caregivers asked for assistance [28]. Authors of another study found that 40% of the 10 to 14-year-olds knew their HIV status through their caregivers [50], which was similar to findings by Mweemba and colleagues [45]. Most studies, however, did not indicate how adolescents came to know their HIV status.

#### Correlates of HIV disclosure

ALWHIV and their caregivers reported to be struggling with disclosure and effects thereof [27, 28, 36, 45, 46, 53]. Caregivers were reported to be reluctant to disclose because they felt that their children could not understand due to their young age [9, 45, 46, 50]. Others failed to disclose HIV status to their children because of the perceived psychological trauma, local norm, anxiety surrounding HIV disclosure, despoliation, unhappiness, abandonment, fear of stigma and discrimination and fear of rejection [27, 28, 36, 45–47, 50, 53]. In addition, disclosure was negatively associated with limited disclosure skills by caregivers and negative attitude by some HIV counsellors [30, 45]. In one study, caregivers revealed that HIV disclosure to a child was much more comfortable if the caregiver was also HIV positive [28].

Knowing one’s own HIV status was more common in female participants (*P ≤* .05) and it increased with age: younger ones were more likely to be unaware of their HIV status (*p* ≤ .001) [30, 41, 60]. Surprisingly, living with a biological parent was related to not knowing HIV status (*p* ≤ .05) [28]. On the other hand, more ALWHIV accessing community healthcare services did not know their HIV status compared to those who had access to hospital services (*p* ≤ .05) [30].

Much as both ALWHIV and caregivers showed positive views after disclosure, some caregivers did not encourage adolescents to talk about their HIV status anywhere [28, 58]. Some participants were against the idea of disclosing their HIV status to their partners because they thought that disclosing to partners rendered them vulnerable to abandonment and exposure [27, 30, 50]. Therefore, ALWHIV prioritised the prevention of pregnancy and stigma, the avoidance of HIV-related stigma and rejection above HIV disclosure in casual relationships [30, 43]. Some ALWHIV concealed their status to maintaining relationships with their partners [30, 46]. In the same way, one study found that the nature, length, or quality of the relationship was an important consideration for disclosure [27]. Still, open family conversation, inquiries on taking medications and threats to stop taking ARVs by ALWHIV, female gender (*p* ≤ .001), desire to promote treatment self-efficacy and facilitating the adoption of safe sexual behaviours among ALWHIV seemed to facilitate HIV disclosure [45, 52]. Notably, disclosure had both negative and positive outcomes. Positive outcomes included opportunities for accessing adherence support, psychosocial support from family members and peers [9, 27, 41, 43, 46, 47, 52]. Conversely, anxiety, anger, pain, confusion, depression, blaming self, strained sexual relationship and rejection followed disclosure [18, 27, 28, 43, 46]. Psychosocial support, through support groups, was commonly mentioned as a significant outcome of disclosure because it provided a sense of belonging and normalcy, although others felt that enlarging the circle of people knowing their HIV status increased the chances of accidental disclosure [27, 31, 41, 43, 46, 52].

## Discussion

This systematic review identified 36 articles related to behaviours among ALWHIV in SSA. We classified the behaviours into four themes, namely ART non-adherence, sexual risk behaviour, alcohol and substance abuse, and HIV disclosure. This systematic review found that many ALWHIV were engaging in risky health behaviours because of: stigma and discrimination; lack of or poor disclosure of HIV status to adolescents; distance to ART clinics; insufficient resources including knowledge; lack of social support, and some inert factors (age and gender). Our discussion suggests solutions to promote adoption of health behaviours among ALWHIV. All the studies (*n* = 36) researched adolescents’ health behaviours and two of them were interventional studies that evaluated behavioural outcomes after tests. Few studies incorporated ALWHIV’s significant others within family and social circles. Similarly, some, studies (15) were qualitative in nature, providing statistically unreliable associations of outcomes. Most of the included studies were cross-sectional, which makes it impossible to ascertain the direction of the associations documented. However, as all the studies but one, scored high (≥ 75%) on quality appraisal with 19 of them scoring 100%. The findings can reliably inform ALWHIV education, practice and policy. In addition, only one study [62] recruited more than 1,000 participants, and six studies [18, 27, 30, 32, 35, 36, 41] had a sample size of over 500 participants. The small sample sizes identified in most of the articles included in this review limit the generalizability of the findings to all ALWHIV in SSA region. Further, very few studies or none were conducted in countries with the highest prevalence rates of ALWHIV [15–19] like Malawi, Nigeria, Kenya, Mozambique and Tanzania and only two studies were interventional. There is, therefore, a need to conduct large mixed methods and longitudinal interventional studies with larger sample sizes to examine and enrich the existing evidence on ALWHIV meticulously and to reach a consensus on the inconsistent findings in SSA.

Measurement of behavioural outcomes lacked uniformity across the studies. Most studies used ‘self’ developed tools to measure the defining variables. For example, some studies assessed the use of condoms between married ALWHIVs and their spouses, but other studies looked into participants with other sexual acquaintances, including girlfriend or boyfriend, and one-time partners. In addition, occasions of condom use varied between ‘at first sexual debut’, ‘last time of sex’ or ‘currently’, which makes it difficult to understand the gravity of the situation. Likewise, ART non-adherence was measured by youth adherence to ART, standardised patient medication adherence, and developed questionnaires [29, 31, 51]. All studies used a qualitative approach to assess HIV disclosure outcomes except one, which included an 8-point gradient scale of disclosure levels and depth to assess disclosure to partners [30]. Most studies used a single category or item to measure risky health behaviour and others combined the items falling under ‘risky health behaviour’. For example, two studies assessed sexual risk behaviour or ART non-adherence or both, and alcohol and substance abuse [35, 38]. Other important risk behaviours that have devastating and long-term effects on the health of adolescents including violence and bodily harm, drug use, unhealthy dietary behaviours, and inadequate physical activity were not assessed [24]. Furthermore, only three studies assessed substance abuse and the findings of these studies included only alcohol and tobacco. None of the studies assessed predictors of risky health behaviours, nor did they group the identified risk behaviours into high or low-risk categories.

The articles included in this systematic review have revealed very high rates of ALWHIV engaging in risky health behaviours. To begin with, the reports indicate high incidents of pregnancy that are not intended, with some ALWHIV below the age of 17 falling pregnant. Irrespective of marital status, early childbearing increases the risk of both mothers and their newborn babies. These risks occur in the following ways: 1) there is a 50% higher risk of baby being still born or dying in the first few weeks of birth; 2) higher risk of both spontaneous or induced abortion, which may result in complications, some being fatal; 3) possible disability caused to both mother and baby; and 4) possible negative socioeconomic consequences such as dropping out of school, unemployment, dependent on family, and the burden of taking care of the baby [63–65]. For these reasons, an ALWHIV (who already faces health risks) who falls pregnant is at higher risk of mortality and morbidity. In addition, ALWHIV reportedly had a low uptake rate of PMTCT services. This could be because ALWHIV need sexual and reproductive health services to be youth friendly and free of the stigma associated with HIV and fertility from health workers [33, 34, 66]. Three SSA studies found that negative clinical experiences, long queues, overcrowded waiting areas, anger, and judgemental staff may also be a barrier to ALWHIV visiting youth reproductive health clinics for services, including information [30, 34, 39]. ALWHIV need these services to acquire information that will help them make an informed decision about protecting themselves and others from HIV infection and reinfection. Further, another study found that it was difficult for an adolescent to access and utilise available HIV services fully, which is a significant problem in the coming era of universal treatment for people with HIV [67]. This could be improved by reducing stigma toward ALWHIV through public sensitisation, changing attitudes of health workers through sensitisation and reinforcement (negative and positive) of favourable behaviour towards ALWHIV, and the development of policies that promote a safe environment to ALWHIV and their families. Some countries like Ghana and Thailand are already practising this and it has proved to be effective [68, 69]. Although many ALWHIV were reported to engage in sex outside marriage, contradictory data from the included studies about gender and sexual activities could be the result of using different measuring tools and study designs, and potential confounding factors. However, this systematic review reveals the need to develop behavioural interventions targeting the reduction of risky health behaviours among ALWHIV. Only two interventional studies for this age group were found. Future studies should examine same sex and transgender sexual practices as emerging trends, as well as reasons why ALWHIV fail to adopt safe sex practice.

Our study found that many adolescents reported financial problems and distance from a clinic as barriers to ART adherence, which is similar to findings of multiple studies in a report by WHO [70]. It is commendable that countries have scaled up ART services, but many adolescents still live at a considerable distance from hospitals. Bringing ART closer to adolescents can have positive outcomes. The introduction of mobile ART clinics in remote communities would help to reduce that burden of travelling a great distance to access care [71]. It is not surprising that while the rate of new infections because of mother to child transmission of HIV is decreasing due to effective ART medication, the rate of new infection is increasing among adolescents in SSA because of their engagement in risky sexual behaviours [3, 7]. It is therefore essential to encourage parents and health care workers to be receptive and open with adolescents in discussing sexual issues. Integrating ART services into youth reproductive health services and support groups may reduce the number of missed visits to clinics to collect ART, and improve uptake of sexual and reproductive services, including condoms and reproductive health information. It is, therefore, important to assist adolescents to adhere to ART at all costs because WHO recommends a high level of adherence in order to: (1) suppress viral replication and improve immunological and clinical outcomes; (2) decrease the risk of developing ARV drug resistance; and (3) reduce the risk of transmitting HIV to others [70]. However, this systematic review was hindered by the lack of uniformity in assessing ART non-adherence across the studies. For example, most studies relied on self-reports of ART adherence for their data where they asked participants if they had missed ART in past time intervals of three days, week or a month, which has a recall bias especially in younger adolescents and long-time periods required to recall (like one month).

Although HIV disclosure is relatively not well elucidated and documented, it is a crucial issue in ALWHIV and needs immediate attention. Studies in this systematic review have associated HIV disclosure with health behaviours and quality of life as such; we recommend that health workers, parents and policymakers should prioritise HIV disclosure in the management of ALWHIV. It is therefore essential to include HIV disclosure in services offered to adolescents to equip them with knowledge and skills regarding disclosure of their HIV status to important people outside the family circle such as teachers and intimate friends, who are likely to support them to meet the demands of living with HIV. In addition, HIV disclosure has remarkably reinforced ART adherence, which promotes viral suppression [50, 72–74], safe sex practices [50, 75], HIV knowledge, and self-care and responsibility for HIV treatment and care [76]. Likewise, it is also important to encourage ALWHIV to join peer support groups once they know their HIV status. Peer support groups assist ALWHIV to find ways to coexist with their condition, to adhere to ART medication, to be resilient, and to understand that other ALWHIV have similar challenges [77, 78]. In the included studies, HIV disclosure for ALWHIV took place at the home or health facility. It would be beneficial for future studies to assess the preferred place for disclosure by adolescents who are aware of their HIV status, including any associated outcomes to determine the most effective approach to disclosing HIV status to adolescents.

## Conclusion

Previous systematic reviews [79–81] have included a wider age range and populations beyond SSA, which may have very different patterns of HIV transmission. This systematic review included broader and different outcomes from studies that used qualitative, quantitative or mixed methods. The findings of this review show that ALWHIV display behaviours, some of which can have devastating and long-term health effects. We recommend integration of HIV health services for ALWHIV into services that normally have reasonable rates of uptake by adolescents. Some factors that hinder ALWHIV to access care are beyond their control, for example, monetary constraints. We recommend deploying mobile or decentralised HIV clinics to bridge the gap between existing services and ALWHIV who are hard to reach or at inconvenient distances from services. A multidisciplinary approach is needed to meet the unique needs of adolescents in SSA. From this review’s findings, the common factor that appeared to promote safe practices of sexual behaviours, ART non-adherence, alcohol and substance abuse, protective factors and HIV disclosure is positive support from either parents, peers or health workers. Therefore, ALWHIV would benefit from both clinic and peer-based behavioural support intervention to change attitudes of health workers, peers, family and significant others; to support community mobilisation and sensitisation; to promote and empower HIV peer support groups and to developing resources for adolescents that are youth friendly. This calls for more studies with mixed and longitudinal approaches, larger samples, and valid variable standard assessment tools to rigorously investigate and understand the patterns of risky health behaviours among ALWHIV. The studies will also shed more light on inconsistent findings and provide a basis for developing the culturally sensitive and feasible interventions for ALWHIV. Many studies were qualitative, which makes it impossible to determine the power of associations. The existing evidence does not propose sufficient effective interventions to promote safe health behaviours among ALWHIV.

## Additional files

Additional file 1: Search strategy. (DOCX 13 kb)

Additional file 2: Characteristics and quality of studies. (DOCX 22 kb)

Additional file 3: Study outcomes and results. (DOCX 41 kb)
Data used in this systematic review was extracted and summarised from peer reviewed articles. Additional files provided illustrate the summaries of the extracted data including search strategy, characteristics of participants, countries where the studies were conducted, types of the studies, aims of the studies, quality of the studies and study outcomes.

## Abbreviations

AIDS: Acquired immunodeficiency syndrome
ALWHIV: Adolescents living with HIV
ART: Antiretroviral therapy
HIV: Human Immunodeficiency Virus
MMAT: Mixed Method Appraisal Tool
MTCT: Mother to Child Transmission of HIV
SSA: Sub-Saharan Africa
WHO: World Health Organization
