# Supplementary material for: Risky behaviours and their correlates among adolescents living with HIV in sub-Saharan Africa: a systematic review
Source: Reprod Health. 2018 Oct 24;15:180. doi: 10.1186/s12978-018-0614-4 (PMC6201550; doi:10.1186/s12978-018-0614-4)
Supplement: Supplementary file 1 — Search strategy. (DOCX 13 kb) [file 12978_2018_614_MOESM1_ESM.docx]

**Additional file 1: Search strategy**

| **Search 1** | **Search 2** | | **Search 3** | | **Search 4** |
| --- | --- | --- | --- | --- | --- |
| Adolescent OR youth OR young adult OR children,  AND  Living with HIV OR HIV infections OR HIV seropositivity OR HIV OR HIV 1 OR HIV 2 OR Human immunodeficiency virus OR AIDS,  AND  Africa OR Sub-Saharan Africa OR South of the Sahara OR South Africa OR Africa Central OR Africa Eastern OR Africa Western OR Africa Southern  AND  Risk factor OR risky behaviour OR unsafe sex OR sexual behaviour OR risk taking OR alcoholism OR Alcohol drinking OR smoking OR attitude OR practice OR health knowledge OR adolescent health OR adolescent behaviour, | Adolescent OR youth OR young adult OR children,  AND  Living with HIV OR infections OR HIV seropositivity OR HIV OR HIV 1 OR HIV 2 OR Human immunodeficiency virus OR AIDS,  AND  Africa OR Sub-Saharan Africa OR South of the Sahara OR South Africa OR Africa Central OR Africa Eastern OR Africa Western OR Africa Southern  AND  HIV disclosure OR truth disclosure | Adolescent OR youth OR young adult OR children,  AND  Living with HIV OR HIV infections OR HIV seropositivity OR HIV OR HIV 1 OR HIV 2 OR Human immunodeficiency virus OR AIDS,  AND  Africa OR Sub-Saharan Africa OR South of the Sahara OR South Africa OR Africa Central OR Africa Eastern OR Africa Western OR Africa Southern  AND  Antiretroviral therapy adherence OR ART adherence OR adherence OR medication adherence OR patient compliance OR highly active antiretroviral therapy OR anti-retroviral agents | | Adolescent OR youth OR young adult OR children,  AND  Living with HIV OR HIV infections OR HIV seropositivity OR HIV OR HIV 1 OR HIV 2 OR Human immunodeficiency virus OR AIDS,  AND  Antiretroviral therapy adherence OR ART adherence OR adherence OR medication adherence OR patient compliance OR Highly active antiretroviral therapy OR anti-retroviral agents OR  Risk factor OR risky behaviour OR unsafe sex OR sexual behaviour OR risk taking OR alcoholism OR Alcohol drinking OR smoking Attitude OR practice OR health knowledge OR adolescent health OR adolescent behaviour,  OR  HIV disclosure OR truth disclosure  AND  Angola OR Benin OR Botswana OR Burkina Faso OR Burundi OR Cameroon OR Cape Verde OR Central African Republic OR Chad OR Comoros OR Congo Brazzaville OR Congo Democratic Republic OR Côte d'Ivoire OR Djibouti OR Equatorial Guinea OR Eritrea OR Ethiopia OR Gabon OR The Gambia OR Ghana OR Guinea OR Guinea-Bissau Or Kenya OR Lesotho OR Liberia OR Madagascar OR Malawi OR Mali OR Mauritania OR Mauritius OR Mozambique OR Namibia OR Niger OR Nigeria OR Rwanda OR Senegal OR Sierra Leone OR Somalia OR South Africa OR Sudan OR Swaziland OR Tanzania OR Togo OR Uganda OR Zambia OR Zimbabwe | |
