# Supplementary material for: Risky behaviours and their correlates among adolescents living with HIV in sub-Saharan Africa: a systematic review
Source: Reprod Health. 2018 Oct 24;15:180. doi: 10.1186/s12978-018-0614-4 (PMC6201550; doi:10.1186/s12978-018-0614-4)
Supplement: Supplementary file 2 — Characteristics and quality of studies. (DOCX 22 kb) [file 12978_2018_614_MOESM2_ESM.docx]

**Additional file 2:** **Characteristics and quality of studies**

| **Author & year** | **Study purpose/Objective** | **Participants and**  **Age** | **Setting** | **Country** | **Sample size** | **Study design & methods** | **MMAT score** |
| --- | --- | --- | --- | --- | --- | --- | --- |
| Ankrah et al., 2016 | To identify facilitators of and barriers to antiretroviral treatment adherence among adolescents in Ghana. | ALWHIV  12-19 years old  (M^age^ 16.3y) | HIV clinic | Ghana | 19 ALWHIV | Cross-sectional qualitative: SSI | 100% |
| Cluver et al., 2016 | To examine associations of 10 social protection provisions with adherence in a large community-based sample of HIV-positive adolescents. | ALWHIV  10-19 years old  (M^age^ 13.8) | Community and HIV clinic | South Africa | 1059 ALWHIV | Cross-sectional-quantitative | 100% |
| Kawuma et al., 2014 | To describe how the experience of life-long HIV and ART interplays with everyday life for young adolescents within the different care environments such as the home, in the clinic and at school. | ALWHIV  11-13 years old  (M^age^ not given) | HIV clinics | Uganda | 26 ALWHIV | Cross sectional Qualitative: interviews | 75% |
| Denison et al., 2015 | To explore ART adherence from the perspectives and experiences of older ALHIV (aged 15-18) and their adult caregivers in Zambia. | ALWHIV 15-18 years old and caregivers  (M^age^ not given) | HIV clinic | Zambia | 32 ALWHIV & 23 caregivers | Cross sectional Qualitative: interviews | 75% |
| Nachega et al., 2009 | To determine adherence to and effectiveness of ART in adolescents versus adults in Southern Africa. | ALWHIV  11-19 years old  (M^age^ 13.8) | HIV clinic | Southern Africa | 154 ALWHIV & 7,622 HIV+ adults | Observational cohort study | 75% |
| Bermudez et al., 2016 | To examine whether differential adherence outcomes are associated with specific economic and social attributes among Ugandan adolescents living with HIV. | ALWHIV  10-16 years old  (M^age^ 12.4) | HIV clinic | Uganda | 702 ALWHIV | Retrospective cohort: interviews | 100% |
| Mahvu et al., 2013 | To come up with a description of internal and external life circumstances of HIV positive young people. | ALWHIV  15-18 years old (Median ^age^ 14) | Africaid HIV support groups | Zimbabwe | 229 ALWHIV | Mixed methods | 100% |
| Enimil et al., 2015 | To examine the challenges and protective factors for adolescents living with HIV within the sociocultural context of Ghana. | ALWHIV  12-19 years old (M^age^ 15.5) | HIV clinic | Ghana | 40 ALWHIV | Mixed methods | 75% |
| Mupambireyi et al., 2014 | To explore peer social support experiences of HIV-perinatally infected children in Harare, Zimbabwe. | ALWHIV  11-13 years old (M^age^ 12.0) | Community HIV support groups | Zimbabwe | 26 ALWHIV | Cross sectional Qualitative  IDIs, FGDs | 100% |
| Thupayagale-Tshweneagae 2010 | To describe adolescents’ perceptions in Botswana on the behaviours that HIV-positive adolescents use in dealing with stigmatization. | ALWHIV  13-19 years old (M^age^ not given) | Day care centres | Botswana | 18 ALWHIV | Exploratory descriptive qualitative: FGDs | 75% |
| Cluver et al., 2015 | To examines associations between adolescent knowledge of HIV-positive status and ART-adherence in South Africa. | ALWHIV  10-19 years old (M^age^ 14.2) | Health facilities | South Africa | 684 ALWHIV | Cross-sectional: mixed methods Interviews, FGDs | 100% |
| Abubakar et al., 2016 | To investigate psychosocial challenges faced by HIV infected adolescents on the Kenyan coast. | ALWHIV  12-17 years old  (M^age^ 14.5) | HIV clinics on health centres | Kenya | 44 ALWHIV | Cross- sectional qualitative: IDIs & SSI | 75% |
| Ndiaye et al., 2013 | To determine the level of ART adherence and predictors of non-adherence among HIV infected adolescents. | ALWHIV  13-18 years old (Median ^age^ 15.0) | HIV Clinic | Botswana | 82 ALWHIV | Cohort quantitative: interviews | 100% |
| Madiba & Mokgatle, 2016 | To explore how adolescents with PAH experience living with HIV and examined their perceptions and experiences regarding disclosure and onward self-disclosure to friends and sexual partners. | ALWHIV  14-18 years old (M^age^ 15.5) | HIV clinic | South Africa | 37 ALWHIV | Qualitative exploratory: IDIs | 75% |
| Mburu et al., 2014 | To explore the disclosure of HIV status to adolescents living with HIV; adolescents’ disclosure of their status to others; and the impact of both forms of disclosure on adolescents. | ALWHIV 10-19 years old, parents & healthcare workers (M^age^ 16.8) | HIV clinic, community & youth centres. | Zambia | 58 ALWHIV | Cross sectional qualitative: SSIs &FGDs | 75% |
| Mandalazi et al., 2014 | To explore potential factors that challenge parents and guardians when informing their perinatally HIV-infected child about the child’s HIV status. | Caregivers of ALWHIV 11-14 year (M^age^ not given) | HIV clinic | Malawi | 20 caregivers | Cross sectional: qualitative, interviews | 50% |
| Lawan et al., 2015 | To determine the awareness of HIV status and risk factors for HIV transmission among HIV-positive adolescents, and how these impact their behaviour. | ALWHIV 10-19 years old (M^age^ 14.9) | HIV clinic | Nigeria | 400 ALWHIV | Cross sectional quantitative: interviews | 100% |
| Mweemba et al., 2015 | To understand factors that facilitate or inhibit caregiver’s ability to disclose the HIV status of adolescents aged 10–15 years. | Caregivers and key informants of ALWHIV 10-15 years old (M^age^ 11.9) | HIV clinic | Zambia | 6 key informants & 30 caregivers | Cross sectional Qualitative: IDIs | 75% |
| Toska et al., 2015 | To identify whether knowledge of HIV-status by HIV-positive adolescents and partners was associated with safer sex. | ALWHIV 10-19 years old (M^age^ 14.3) | HIV clinic | South Africa | 858 ALWHIV | Cross sectional: mixed methods, interviews, FGDs, observation | 100% |
| Bhana et al., 2014 | To report the short-term impact of the VUKA family program on a range of psychosocial variables for ALWHIV and their caregivers. | ALWHIV 10-19 years old  (M^age^ not given) | HIV clinic | South Africa | 65 ALWHIV and their families | Randomised trial: interviews, FGDs | 75% |
| Vreeman et al., 2015 | To describe life experiences of ALWHIV | ALWHIV 10-14 years old (M^age^ 12.3) &caregivers | HIV clinic | Kenya | 285 caregiver–child dyads | Cross sectional quantitative: interviews | 100% |
| Nöstlinger et al., 2014 | To investigate the psychological and  social factors influencing self-disclosure of own HIV status to peers. | ALWHIV  13-17 years old (M^age^ 14.6) | HIV clinic | Uganda and Kenya | 582 ALWHIV | Cross sectional quantitative: face to face interviews | 100% |
| Bernays et l., 2015 | To examine children’s experiences of living with HIV on treatment. | ALWHIV 11-13 years old (M^age^ not given) | HIV clinic | Zimbabwe and Uganda | 104 ALWHIV | Prospective cohort Qualitative: IDIs & FGD | 75% |
| Hodgson et al., 2012 | To explore and document the informational, psychosocial, sexual and reproductive health (SRH) needs of adolescents living with HIV and identify gaps between needs and existing services. | ALWHIV 10-19 years old  (M^age^ not given) | HIV clinic | Zambia | 111 ALWHIV and 59 key informants | Explorative qualitative: SSIs and FGDs | 100% |
| Petersen et al., 2010 | To develop an understanding of the psychosocial challenges as well as protective influences promoting socio-emotional coping in HIV+ adolescents to inform mental health promotion and HIV prevention programming for this population in South Africa. | Caregivers & ALWHIV  14-16 years old (M^age^ not given) | HIV clinic | South Africa | 25 ALWHIV &15 caregivers | Cross sectional Qualitative: IDIs | 100% |
| Kim et al., 2015 | To identify contributory/protective factors associated with depression in Malawian adolescents 12–18 years old living with HIV. | ALWHIV 12-18 years old (M^age^ 14.5) | HIV clinic | Malawi | 562 ALWHIV | Cross-sectional quantitative: structured interviews | 75% |
| Mbalinda et al., 2015 | To assessed factors associated with better HRQoL in perinatally HIV -infected adolescents. | ALWHIV 10-19 years old  (M^age^ 16.2) | HIV clinic | Uganda | 572 ALWHIV | Cross sectional quantitative: interviews | 100% |
| Mbalinda et al 2015 (a) | To explore the correlates of ever had sex among perinatally HIV-infected (PHIV) adolescents | ALWHIV 10-19 years old  (M^age^ 16.2) | HIV clinic | Uganda | 624 ALWHIV | Cross sectional quantitative: interviews | 100% |
| Birungi et al., 2009 | To examine the sexual expressions and experiences, preventive practices and implication of these of HIV/AIDS programs of adolescent born with HIV. | ALWHIV 15-19 years old  (M^age^ 17) | HIV clinic & support programs/groups | Uganda | 732 ALWHIV | Mixed methods: structured and extended interviews, FGDs | 100% |
| Senyoni et al., 2012 | To explore the effects of the CBT group counselling intervention, the independent variable IV, on the dependent variables of participant’s transmission risk behavior, depression levels, anxiety levels and alcohol use rates. | ALWHIV 12-18 years old  (M^age^ 15.19) | Health facility | Uganda | 171 ALWHIV | experimental pretest-posttest randomized controlled trial: interviews | 75% |
| Forrest et al., 2009 | To characterize the attitudes towards HIV and childbearing among the adolescent community. | ALWHIV 16-18 years old  (M^age^ 16.8) | HIV clinic | South Africa | 19 ALWHIV | Cross sectional Qualitative: FGDs | 100% |
| Obare et al., 2012 | To examines the factors associated with experiencing unintended pregnancies, poor birth outcomes, and post-partum contraceptive use among HIV-positive female adolescents. | ALWHIV 15-19 years old  (M^age^ not given) | HIV clinic | Kenya | 394 ALWHIV | Cross sectional: structured interviews | 75% |
| Vujovic et al., 2014 | To examine programmatic approaches to the sexual and reproductive health of very young adolescents. | ALWHIV 10-14 years old  (M^age^ not given) | HIV clinic | South Africa | 27 HIV clinic | Qualitative: FGDs | 100% |
| Obare et al., 2010 | To compare the sexual and reproductive experiences and intentions of HIV+ adolescents | ALWHIV 15-19 years old  (M^age^ 16.7) | HIV clinic and communities | Uganda | 732 ALWHIV | Mixed methods | 75% |
| Birungi et al., 2011 | To examine maternal health care utilization. | ALWHIV 15-19 years old  (M^age^ not given) | HIV clinic | Kenya | 393 ALWHIV | Mixed methods | 75% |
| Arikawa et al., 2016 | To estimate the incidence of pregnancy and its associated factors. | Female ALWHIV 10-19 years old  (median age 12.8) | HIV clinic | Coˆte d’Ivoire | 266 ALWHIV | Retrospective cohort study: Interviews and medical records | 100% |

HIV=human immunodeficiency virus, ALWHIV= Adolescents living with HIV, FGD= focus group discussions, IDI= In-depth interviews, SSI= semi-structured interview, MMAT= mixed method appraisal tool, ART= antiretroviral therapy, M^age^= mean age.
