# Supplementary material for: Risky behaviours and their correlates among adolescents living with HIV in sub-Saharan Africa: a systematic review
Source: Reprod Health. 2018 Oct 24;15:180. doi: 10.1186/s12978-018-0614-4 (PMC6201550; doi:10.1186/s12978-018-0614-4)
Supplement: Supplementary file 3 — Study outcomes and results. (DOCX 41 kb) Data used in this systematic review was extracted and summarised from peer reviewed articles. Additional files provided illustrate the summaries of the extracted data including search strategy, characteristics of participants, countries where the studies were conducted, types of the studies, aims of the studies, quality of the studies and study outcomes. [file 12978_2018_614_MOESM3_ESM.docx]

**Additional file 3: Study outcomes and results**

| **Author & year** | **Study outcomes** | **Results** |
| --- | --- | --- |
| Ankrah et al., 2016 | Drug adherence | - Predicted probability of past-week nonadherence without social protection was 54% - **Facilitators**: Support from health care providers and parents, adolescents’ knowledge of disease, self-motivation, perceived positive outcomes - **Barriers:** Forgetfulness, perceived stigmatization due to disclosure, financial barriers, and adverse effects of ART |
| Cluver et al., 2016 | Drug adherence | - **Negative correlates**: Current opportunistic infections (B 0.27, *p* = .005, SD .09) and increased rates of detectable viral load (aOR 1.98, 95% CI:1.13–4.45, *p* < .05) - **Positive correlates**: Sufficient food (aOR 0.57, 95% CI: 0.42–0.76, *p* < .001); attending an HIV support group (aOR 0.60, 95% CI: 0.40–0.91, *p* < .05) and high parental supervision/ monitoring (aOR 0.56, 95% CI: 0.43–0.73, *p* < .001) |
| Kawuma et al., 2014 | Drug adherence | - **Facilitators:** Fear of being scolded by guardians and health care workers - **Barriers:** Lack of HIV status dislosure, lack of food, side effects, fear of being seen by others, lack of time |
| Denison et al., 2015 | Drug adherence | - More than a quarter of respondents experienced ART interruptions ranging from one day to six months - **Facilitators**: Family support, youth support groups, prolonged illness before ART initiation and desire to live longer - **Barriers**: Fear of unintentional disclosure, away from home, side effects, not wanting to take drugs daily and religious healing |
| Thupayagale-Tshweneagae 2010 | Drug adherence | - Adherence was reported as important - Feared disclosure and kept quite - Reported seeking treatment earlier for common conditions |
| Nachega et al., 2009 | Drug adherence | - A significantly smaller proportion of ALWHIV achieved 100% adherence at each time point (ALWHIV: 20.7% at 6 months, -14.3% at 12 months, 6.6% at 24 months; adults: 40.5%, 27.9%, and 20.6% at each time point, respectively; *p* < 0.01) - 100% 12-month adherence was associated with virological suppression at 12 months, regardless of age - ALWHIV achieving virological suppression had significantly shorter time to viral rebound (aOR 2.03; 95% CI:1.31–3.13; *p* < 0.003) - ALWHIV were less likely to experience long-term immunologic recovery despite initial CD4+ T-cell counts comparable to adults - Adolescence was associated with non-ART adherence (*p* <0.01) |
| Bermudez et al., 2016 | Drug adherence | - 62.5% reported to have missed doses - 71% (n=494) of respondents reported optimal adherence to ART, compared to 29% (n=206) - **Facilitators**: Greater asset ownership, (OR 1.69, 95% CI: 1.00-2.85, *p* =.049), greater economic advantage in ownership of household assets, financial savings, and caregiver employment (OR 1.70, 95% CI: 1.07–2.70, *p* = < .05) - **Barriers**: Distance to clinic (OR 1.49, 95% CI: 1.02-2.18, *p* =.04) |
| Mahvu et al., 2013 | Drug adherence | - 21 % of participants reported a 50% adherence, 6% reported 86% adherence, 6% reported a 93% adherence, 4% reported a 96% adherence and 12% reported a 98% adherence - Self-reported adherence was sub-optimal - **Barriers**: Forgetfulness (45%), travelling (23%), concealing from others (22%) and lack of bus fare to collect medication (17%) |
| Enimil et al., 2015 | Drug adherence | - Quantitative analyses’ results suggested that a significant number of participants were not virally suppressed - **Barriers**: Limited social support, concerns about disclosure and HIV-related stigma, limited resources, and lower than expected quality of life |
| Mupambireyi et al., 2014 | Drug adherence | - Children tended to associate not knowing how they got infected with HIV with poor adherence - Teaching helped children to talk more about their personal drug adherence slippages during support group meetings - Perceived stigma |
| Thupayagale-Tshweneagae 2010 | Drug adherence | - All participants reported to be adhering to AIDS therapies and seeking early treatment for common conditions |
| Cluver et al., 2015 | Drug adherence | - Self-reported non-adherence in the past week was 36% - Full adherence was associated with lower likelihood of two or more of concurrent shingles, mouth ulcers or tuberculosis symptoms (OR 0.55; 95% CI: 0.40–0.76, *p* < 0.001) - Adolescent knowledge of their HIV-status doubled the odds of past-week full adherence (OR 2.18; 95% CI: 1.47–3.24, *p* < 0.001) - Full adherence was negatively associated with being older (OR 0.85; 95% CI :0.80–0.91, *p* < 0.001) and longer travel time to the clinic (OR 0.72; 95% CI: 0.57–0.89, *p* < 0.005) - Disclosure prior to age 12 was associated with past-week full adherence (OR 2.07; 95% CI :1.34–5.22, *p* <.005) - Full adherence was negatively associated with longer travel time to the clinic (OR 0.54;95% CI :0.39–0.73, *p*<.001) - Where ALWHIV were threatened with social censure, illness or death should they default from ART, disclosure processes were characterized by anxiety, anger and risks of prompting rebellion through ART defaulting |
| Abubakar et al., 2016 | Drug adherence | - Several health workers and caregivers indicated that it was challenging to ensure medical adherence for ALWHIV under their care |
| Ndiaye et al., 2013 | Drug adherence | - Male gender was associated with ART non-adherence (OR 3.29, 95% CI: 1.13–9.54; *p* = 0.03) - Study participants who self-reported suboptimal ART adherence was more likely to report having ever missed ART doses due to failure to pick up medication at the pharmacy (30.0% versus 9.7%, *p* = 0.03) |
| Madiba & Mokgatle, 2016 | Social disclosure | - 25% ALWHIV had disclosed their HIV status to other people, 10.5% disclosed to a friend or friends 10.5% disclosed to a teacher, 13.5 % indicated they had romantic partners but none had disclosed to their partners - Many thought it was a good thing to disclose - Disclosure event was extremely shocking, hurtful and made them cry - Some reacted with anger, pain confusion and denial - Some were happy with disclosure, learned to accept and move on with normal life after knowing their status - Felt HIV is their secret and there is no need to disclose - Difficult to move on because they were constantly reminded of their status - Most (25) ALWHIV had known about their diagnosis for more than 5 years - Disclosure for most of the ALWHIV was preceded by episodes of serious illness |
| Mburu et al., 2014 | Social disclosure | - **Barriers**: Local norms that deter parents from communicating with their children about sexuality, fear of HIV stigma, and a presumption that ALWHIV would not understand; and fear of rejection - **Facilitators**: Open family conversations - **Impact**: Anxiety, depression, rejection, self-blame and strained relationships. It also allowed ALWHIV to access adherence support and other forms of psychosocial support from family members and peers |
| Mandalazi et al., 2014 | Social disclosure | - Fear of child’s unhappiness after disclosure, making their own status known to more people, and fear of confrontation or creating enmity with their child were barriers to HIV disclosure |
| Lawan et al., 2015 | Social disclosure | - 62.2% of ALWHIV knew their HIV status - The age of 14 years and above was associated with HIV status awareness (z = 11.36, *p* = .0001) - Having at least secondary school level of education was associated with awareness of HIV status (z = 2.78, *P* = .005) - Sex of the clients (X^2^ = 14.9, *p* = 0.0001), and the educational attainment of their mothers (X^2^ = 5.2, *p* = .02) were associated with awareness of HIV status - 48.2% of ALWHIV who knew their HIV status engaged in unprotected sex (*p* =.0001), 19.9% had multiple sexual partners (*p*=0.0001) and had ever used drug/substance (*p* =0.0008), compared to 6%, 4.7% and 0% respectively of those who did not know their status |
| Mweemba et al., 2015 | Social disclosure | - 17 out of 30 (56.7 %) caregivers had disclosed to ALWHIV about their HIV status - Disclosure processes happened either at the home or at the clinic - **Facilitators:** Inquiries on taking medications and threats to stop taking ARVs by ALWHIV, desire to promote treatment self-efficacy and facilitating adoption of safe sexual behaviour among ALWHIV - **Barriers**: Fear of psychological trauma for the ALWHIV, disclosure to other people by ALWHIV which could attract HIV stigmatization for the family and caregivers’, limited disclosure skills by caregivers and negative attitude by some HIV counsellors |
| Toska et al., 2015 | Social disclosure | - 14.9% of the total sample had ever had sex, and 85.1% of sexually active ALWHIV had boy/girlfriend - Knowing one’s status was associated with safer sex (OR = 4.36, CI :1.09–17.47, *p* = .038) - Girls were more likely to report disclosing to their partners (*p ≤* .001) - Knowing one’s HIV-status remained associated with safe sex (OR = 4.36, CI:1.08–17.50, *p* = .038) - ALWHIV who knew their status attempted to use condom with their partners more than those who did not know their status - Disclosure of HIV-positive status to partners rendered ALWHIV vulnerable to abandonment and exposure - The prevention of pregnancy and the avoidance of HIV-related stigma and rejection were prioritised above HIV disclosure, particularly within casual relationships - Participants postponed disclosure until the relationship was stable, concealing their status to maintain sexual desirability, which they feared would be lost through HIV disclosure - The disclosure of HIV status to a partner did not further enable or enhance safe sex negotiation. - Anxiety related to disclosure of HIV status to sexual partners - ALWHIV feared abandonment, despoliation and rejection if they disclosed to their partners - ALWHIV were more committed to keeping their status a secret than health practices for their survival |
| Thupayagale-Tshweneagae 2010 | Social disclosure | - Participants did not disclose their HIV status to friends, partners and family members |
| Mupambireyi et al., 2014 | Social disclosure | - Support groups were considered as a threat of accidental and deductive disclosure by ALWHIV |
| Bhana et al., 2014 | Social disclosure | - Caregivers reported greater change in comfort communicating sensitive topics to their children (*p* < .002) - Caregivers in VUKA experienced less stigma than those in the control group (*p* <.09) - Showed improvements in treatment knowledge (*p* <.08) - Caregiver-child communication improved in youth intervention group (*p* <.09) - ALWHIV in VUKA improved self-concept and future orientation - Vuka improved and strengthened social support for caregivers and children plus new friendships with others of similar condition - Vuka helped caregivers to talk about sensitive topics with their children - Diffusion of the program to other family members - Assisted with caring for the children with HIV |
| Vreeman et al., 2015 | Social disclosure | - Caregivers who had already disclosed to their children had positive views of disclosure - Feeling that ALWHIV were young and could not understand, feared potential psychological trauma, and stigma and discrimination hindered disclosure - Disclosure improved adherence |
| Nöstlinger et al., 2014 | Social disclosure | - Disclosure was positively affected by orphan-hood, older age, self-efficacy, positive self-image, having experienced less public stigmatization and perceived social support |
| Bernays et l., 2015 | Social disclosure | - ALWHIV were not encouraged to talk about their diagnosis anywhere |
| Abubakar et al., 2016 | Social disclosure | - There was partial disclosure to family and peers - ALWHIV preferred to avoid stigma, yet it contributed to anxiety and complicated social relationships - Having a support system eg. a parent or a counsellor, helped them deal with distress quickly - Disclosing their HIV status to people within their immediate environment seemed to be a challenge - ALWHIV and their caregivers believed that disclosure risked them to rejection, isolation, and stigmatisation |
| Hodgson et al., 2012 | Social disclosure | - Social networks had significant impact on management of the disclosure aftermaths - Some participants were angry because they were not told their diagnosis - Stigma led to non-disclosure or delayed disclosure to friends and sexual partners |
| Petersen et al., 2010 | Social disclosure | - **Challenges for ALWHIV**: Dealing with loss of biological parents, coming to terms with their diagnosis, identity difficulties, external stigma and discrimination; and disclosure difficulties - **Facilitators for coping:** Medication, HIV information, a future orientation, financial support and social support - **Challenges for caregivers**: disclosure and lack of financial, family and social support emerged as key challenges |
| Kim et al., 2015 | Social disclosure | - Non-HIV disclosure was associated with depression but was not significant |
| Mbalinda et al., 2015 | Risky sexual behaviours | - Lack of communication with guardians on sexuality (AOR = 0.60, 95 % CI: 0.40–0.89), were associated with poorer mental health outcomes - The ALWHIV with one sexual partner were more likely to present with better mental health (OR: 1.94, 95 % CI :1.00–3.74) compared to those with two or more sexual partners - There were no statistically significant associations between ‘ever had sex, ever been pregnant, condom use, contraceptive use’ and physical or mental health |
| Mbalinda et al., 2015 (a) | Risky sexual behaviours | - 64% had ever been in an intimate relationship - 33% were currently in an intimate relationship - 34% had ever had sexual intercourse and 57% of these were female - Female gender was associated with ever had sexual intercourse (OR = 1.71, 95 % CI =1.21-2.42, *p =.001*) - Age group of 15–19 years was associated with ever had sexual intercourse (OR =12.38, 95 % C I = 5.34-28.7, *p = .001*) - Living alone was associated with ever had sexual intercourse (OR = 5.37, 95 % CI :1.79-16.09) - 10% of adolescent aged 15–19 years had their first sex before the age of 15 - 2.5 % of ALWHIV who ever had sex had used condoms at last sex compared to 97.5 % ALWHIV aged 15–19 years - 16% of those who ever had sex had more than one concurrent sexual partner - 49% of those who ever had sex had been pregnant or made someone pregnant - 64% of those who ever had sex had it in the last six months - 44% of those who ever had sex did not use condoms during and 76 % did not use condoms consistently - 49 % of those who ever had sex had been pregnant or made someone pregnant - 16% of total sample ever had symptoms of sexually transmitted infection |
| Birungi et al., 2009 | Risky sexual behaviours | - 62% of the respondents had ever engaged in either kissing, touching, fondling, masturbation, or fantasizing about love and sex - 58% of boys and 45% of girls were using contraception - 35% and 39% of boys and girls respectively used method at first sex - 89% and 63% of boys and girls respectively had had sex - 53% of adolescents who reported to have had sex never used contraception - Disconnect between the fears of the ALWHIV and their actual preventive practices - Older ALWHIV (those aged 18-19 years) than younger ones (those aged 15-17 years) had engaged in sex *p* <0.05 - More male than female had ever had sex (*p* <0.005) - More boys used contraception in previous relationships - ALWHIV with one sexual partner had better mental health (OR: 1.94, 95 % CI :1.00–3.74) than those with multiple sexual partners |
| Senyoni et al., 2012 | Risky sexual behaviours | - Less number of sexual encounters - Less number of sexual partners - Total score for sexual transmission behaviour for both the experimental and control groups reduced (*p* = 0.017) |
| Forrest et al., 2009 | Risky sexual behaviours | - ALWHIV raised concern over re-infection by HIV positive couples attempting to conceive - Some believed that unprotected sex can re-infect individual with an ARV resistant virus - Lack of youth friendly services and public clinic nurses generated much of the stigma surrounding the issue of HIV and fertility - It was suggested to revise adolescent sexual and reproductive health services to make them more youth-friendly where users could avoid stigma generated by community healthcare workers |
| Obare et al., 2012 | Risky sexual behaviours | - More than 80% of ever pregnant HIV-positive adolescent girls were aged 18–19 years - 24% had been pregnant more than once - 23% of girls were married or living with a man at the time of the interview - 10% of the pregnancies resulted in either miscarriage, stillbirth or abortion - Pregnancies were significantly less likely to be unintended if husband rather than the boyfriend/fiancé or other persons friend/acquaintance/stranger) was responsible (*p* <0.01) - Higher order pregnancies were significantly more likely to result in adverse outcomes compared to lower order ones *(p* <0.01) - Pregnancies were significantly less likely to result in adverse outcomes if they occurred within marital unions (*p* <0.05) - There was no significant difference in the likelihood of post-partum contraceptive use by whether the pregnancy was unintended |
| Vujovic et al., 2014 | Risky sexual behaviours | - Lack of the sexual and reproductive health programs for ALWHIV: girls wanted to learn about pregnancy - Service providers were generally not proactive in addressing the sexual and reproductive health needs of ALWHIV - Poor parent–child communication: ALWHIV feared that seeking guidance or advice could be interpreted that they were either sexually active or planning to - Generally embarrassed to talk about sexual matters - ALWHIV believed that caregivers gave wrong information on purpose to deter them from sexual activity - Turned to their peers, relied on the media and the internet and built knowledge in life - Ignorant on how to use a condom |
| Toska et al., 2015 | Risky sexual behaviours | - Healthcare workers used adolescents’ fear of exposure to both threaten and motivate ALWHIV into changing risky behaviours. - Nurses openly accused ALWHIV of recklessness and purposeful HIV transmission in the presence of other healthcare workers and patients - Healthcare workers expressed anger towards the perceived recklessness of their adolescent patients - Sexual risk-taking was widely acknowledged and censured by healthcare workers, who treated STIs cases with frustration and bewilderment - Healthcare workers used blame and humiliation strategically to discourage risky behaviours - Condom use was still inconsistent and subject to difficult negotiations - Girls were more likely to have ever had sex (*p* ≤ .001) - More females had had sex than males (*p* <0.001) - More males than females had abstained or used a condom (*p* <0.05) - More females than males engaged in unprotected sex (*p* <0.05) |
| Obare et al., 2010 | Risky sexual behaviours | - A significantly higher proportion of ALWHIV who knew their HIV status reported using a preventive method at first intercourse (*p* <0.05) ever use of a contraceptive method (*p* <0.05, current use of a modern method of contraception (*p* <0.01), and current condom use (*p* <0.01) than their counterpart who did not know their status - There was a significantly higher proportion of female respondents who did not know their HIV status than their counterparts who were HIV-positive - Male ALWHIV were less likely to use contraception/ condoms compared to females (*p* <0.005) - Married or living together had ever used contraception method (*p* <0.005) |
| Birungi et al., 2011 | Risky sexual behaviours | - 74% of pregnancies occurred within unmarried ALWHIV - 71% pregnancies occurred to those with primary level education - 79% pregnancies happened to ALWHIV who had first pregnancies before age of 18 - People other than husbands were responsible for 75% of pregnancies - 74% of pregnancies were unintended - Pregnancy occurrence increased with age - ALWHIV received prenatal care in 84% of their pregnancies - Proportion of pregnant ALWHIV who received PMTCT services was lower than those who received prenatal care (67% vs. 84%; *p* <.01) - Most of the pregnancies resulted in a live birth (85%) - Receiving prenatal care was more likely if husband was responsible for pregnancy - ALWHIV were significantly less likely to have received PMTCT services for higher-order pregnancies than for lower-order pregnancies - ALWHIV were more likely to receive PMTCT services if they had made at least four prenatal care visits than if not - ALWHIV had significantly higher odds of receiving skilled assistance if they had made at least four prenatal care visits than less - The proportion of pregnancy outcomes for which ALWHIV received skilled attendance was significantly lower for miscarriages, stillbirths or abortion than for full-term or preterm live births (20% vs. 79% and 78%, respectively (*p* <.01) |
| Arikawa et al., 2016 | Risky sexual behaviours | - There were 17 incident pregnancies (2 recurring) - 28.5% had (induced or spontaneous) - Median age at first pregnancy was 17.7 years - Maternal death was common event (one in two girls) - Pregnancy occurrence was frequent in girls who had lost mothers - Incidence of pregnancy increased with age |
| Kim et al., 2015 | Alcohol, Drug and Substance use | - 2.7% of participants took alcohol once or many times for the past month - Alcohol use was associated with not having a boyfriend/girlfriend with depression (OR 0.05, 95% CI: 0.31-0.92, *p* =0.02) - Never using alcohol was associated with depression (*p* =0.04) |
| Mbalinda et al., 2015 | Alcohol, Drug and Substance use | - Non-smoking ALWHIV were nearly twice likely (OR 1.70, 95 % CI: 1.09–2.74) to present with better physical health than ALWHIV who were smoking |
| Senyoni et al., 2012 | Alcohol, Drug and Substance use | - Alcohol consumption decreased after intervention |

CI= Confidence interval, HR=Hazard Ratio, OR= Odds Ratio, PMTCT= prevention of mother to child transmission, SD= standard deviation, P=power of significance, HIV= human immunodeficiency syndrome, ART- antiretroviral therapy, X^2^= Chi square, Z=standard score, ALWHIV= adolescents living with HIV
